# Effect of a Smartphone Application on Physical Activity and Weight Loss After Bariatric Surgery—Results from a Randomized Controlled Trial

**DOI:** 10.1007/s11695-023-06753-6

**Published:** 2023-07-27

**Authors:** Stephanie E. Bonn, Mari Hult, Kristina Spetz, Helén Eke, Ellen Andersson, Mikael Wirén, Marie Löf, Ylva Trolle Lagerros

**Affiliations:** 1 Clinical Epidemiology Division, Department of Medicine (Solna), Karolinska Institutet, Maria Aspmans Gata 30A, SE-171 64 Stockholm, Sweden; 2 Unit of Gastroenterology, Department of Medicine (Huddinge), Karolinska Institutet, Stockholm, Sweden; 3 Department for Upper GI Diseases, Karolinska University Hospital, Stockholm, Sweden; 4 Department of Surgery, Linköping University, Norrköping, Sweden; 5 Department of Biomedical and Clinical Sciences, Linköping University, Norrköping, Sweden; 6 Department of Surgery, Ersta Hospital, Stockholm, Sweden; 7 Department of Health, Medicine and Caring Sciences, Division of Society and Health, Linköping University, Linköping, Sweden; 8 Department of Biosciences and Nutrition, Karolinska Institutet, Stockholm, Sweden; 9 Center for Obesity, Academic Specialist Center, Stockholm, Sweden

**Keywords:** Bariatric surgery, Intervention, mHealth, Obesity, Physical activity

## Abstract

**Purpose:**

Ways to motivate and support patients in being physically active after bariatric surgery are needed. This trial was aimed at evaluating the effect of using a smartphone application targeting physical activity during 12 weeks on moderate-to-vigorous physical activity (MVPA, primary outcome) and secondary outcomes of inactivity, light physical activity (LPA), body mass index (BMI), and percent total weight loss (%TWL) after bariatric surgery.

**Materials and Methods:**

Data from a randomized controlled trial comprising 146 patients (79.5% women) undergoing bariatric surgery was analyzed. Mean age and BMI pre-surgery were 40.9 years and 40.5 kg/m^2^, respectively. Participants were randomized 1:1 to an intervention or a control group. Physical activity and body weight were objectively measured at baseline pre-surgery and post-surgery follow-ups after 6 weeks (weight only), 18 weeks, 6 months, and 1 year. Linear mixed models were fitted to assess longitudinal differences in outcomes between the groups.

**Results:**

A significant effect of the intervention (group-by-time interaction 16.2, 95% CI 3.5 to 28.9) was seen for MVPA at 18 weeks; the intervention group had increased their MVPA since baseline, while the control group had decreased their MVPA. The control group had lowered their BMI approximately 1 kg/m^2^ more than the intervention group at follow-up after 18 weeks and 12 months, yet, mean BMI did not differ between the groups. No intervention effect was seen on inactivity, LPA, or %TWL.

**Conclusion:**

Our results indicate that use of a smartphone application targeting physical activity may have the potential to promote short-term MVPA post bariatric surgery.

**Trial Registration:**

Clinicaltrials.gov: NCT03480464

**Graphical Abstract:**

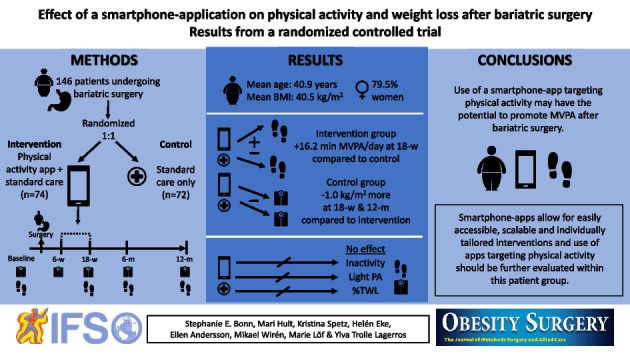

## Introduction

Lifestyle changes, including increased physical activity, are important to optimize post-operative outcomes of bariatric surgery. Positive relationships between post-operative exercise and weight loss have been shown in systematic reviews of observational studies [1–3] and more recently in controlled trials [4], although contradictory results showing no effect have also been published [5]. Exercise post bariatric surgery has also been shown to improve physical fitness and muscle strength [4, 6]. Yet, patients experience a lack of support from health care regarding physical activity after bariatric surgery [7].

While studies using self-reported measures of physical activity have suggested that patients increase their physical activity the first year after surgery [8, 9], two studies using objective methods did not confirm these findings [10, 11]. However, in a meta-analysis of physical activity in patients undergoing bariatric surgery, Adil et al. [12] showed that while no statistically significant improvement in objectively measured physical activity was seen during the first 6 months after surgery, significant improvements were seen during longer follow-ups after 6–12 months and 12–36 months. Targeting physical activity preoperatively by individual face-to-face counseling sessions has also been seen to favor physical activity levels post surgery [13].

Interventions targeted towards increasing physical activity in patients following bariatric surgery show mixed results, but seem to facilitate positive changes according to a recent review [14]. However, no mHealth (mobile health) intervention was included. mHealth, including use of smartphone applications, can be used to deliver interventions directly to patients [15]. As smartphones are an integral part of life today for many people, use of different applications has made it possible to engage patients in self-care at their own convenience [16]. Use of smartphone applications to support weight loss has shown positive results in both the general population and among adults with overweight or obesity [17]. The effect of smartphone applications to increase physical activity is less clear [18, 19], and studies evaluating the effect of applications specifically developed to target physical activity after bariatric surgery are lacking. Nevertheless, digital solutions developed to support patients after bariatric surgery are sought after [20].

The aim of this study was to evaluate the effect of physical activity intervention delivered via the PromMera smartphone application, on objectively measured moderate-to-vigorous physical activity (primary outcome) and inactivity, light physical activity, body mass index, and percent total weight loss (secondary outcomes) after bariatric surgery.

## Method

The randomized controlled trial was designed to investigate the effect of a smartphone application promoting physical activity and supporting intake of vitamin and mineral supplementation after bariatric surgery. The trial design [21] and results from the evaluation of vitamin and mineral supplementary intake [22] have been described in detail previously. The trial was registered at www.ClinicalTrials.gov (NCT03480464).

### Study Participants

Patients referred to the surgical outpatient clinic for bariatric surgery at a county hospital in Sweden from Nov 2017 until May 2019 were eligible for inclusion. All patients fulfilled the indication for surgery (i.e., body mass index (BMI) ≥ 35 kg/m^2^). Inclusion criteria for the trial were being accepted for gastric bypass or sleeve gastrectomy, age 18–60 years, ability to read and understand Swedish, and access and ability to handle a smartphone. Exclusion criteria were disability preventing daily walking.

**Fig. 1:**
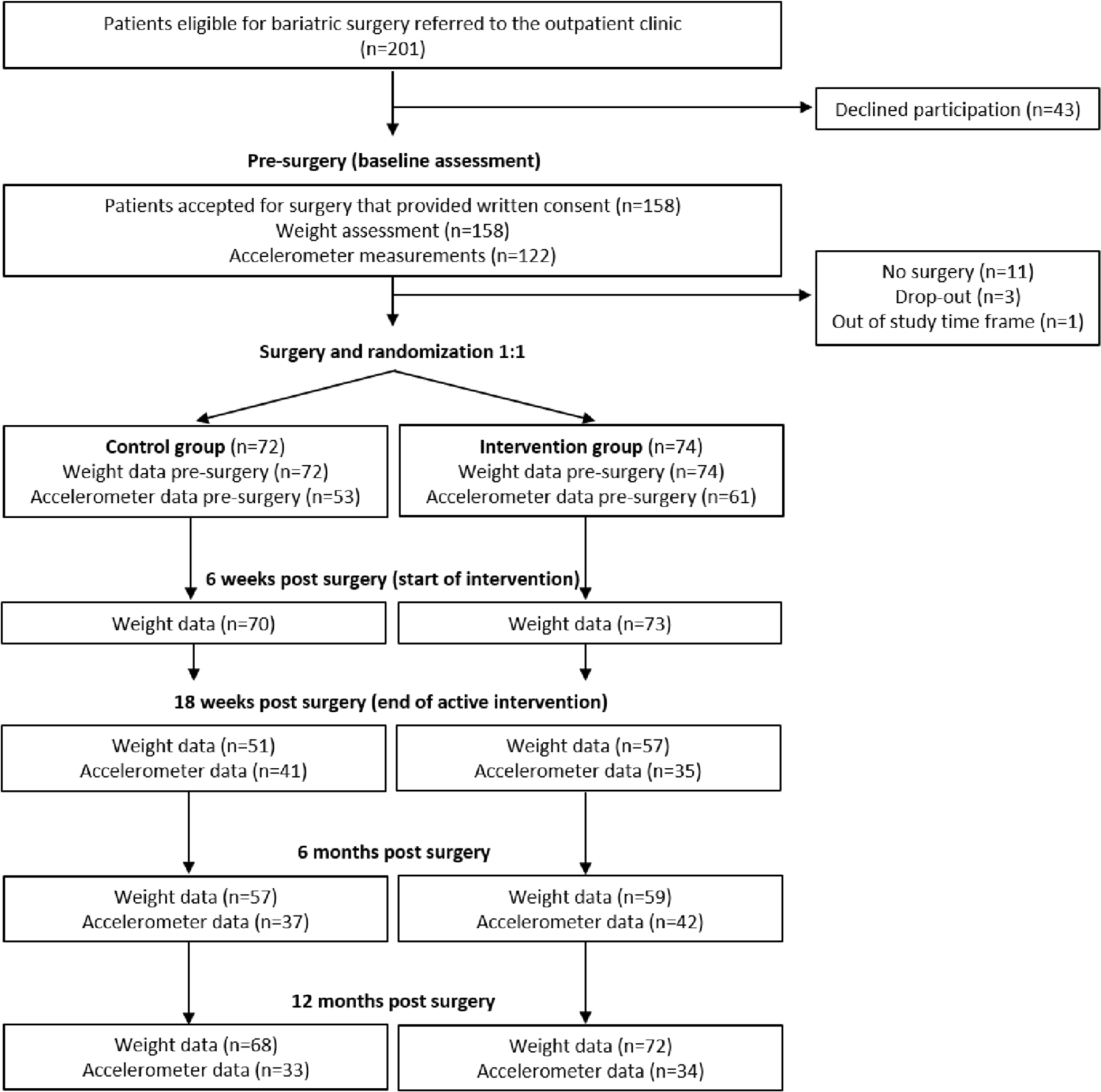
Flow of participants and available data at baseline and follow-ups during the PromMera study

Patients eligible for surgery were invited to a group meeting and an individual appointment at the outpatient clinic as part of standard care, during which they received information about the surgical procedure and the study. Patients that were accepted for surgery were contacted by a nurse and given additional oral information about the study. Those who wanted to participate gave their oral consent during the call and were thereafter sent an informed consent form, the baseline study questionnaire, and an accelerometer. The signed consent, filled out questionnaire, and the accelerometer were returned to study personnel in a pre-paid envelope before surgery. Participants were randomized after surgery, independent of the surgical procedure that had been performed. The type of surgical procedure selected was decided up on as part of standard care. At the participating hospital, gastric bypass was the standard procedure, and sleeve gastrectomy was offered only to patients who were considered not suited for gastric bypass because of extensive adhesions in the lower abdomen, a history of Crohn’s disease, or duodenal or bile duct disease in need of endoscopic surveillance. Additional study questionnaires and accelerometers were sent out to participants 18 weeks post surgery and again after 6 and 12 months of follow-up. Figure 1 shows the flow of participants and available data at baseline and follow-ups.

### Randomization

Participants were randomly allocated 1:1 to the intervention or control group using block randomization. Women were randomized in blocks of four and men in blocks of two due to the majority of bariatric surgery patients being women. Participants were informed about their allocation at the 6-week post-surgery appointment.

### Physical Activity Intervention

Participants in the intervention group were given access to the smartphone application at their post-operative appointment 6 weeks after surgery. They received a personal login and were asked to use the application during the following 12 weeks. Every Monday, participants were asked to set a weekly physical activity goal of 100, 150, 210, or 250 min of moderate-to-vigorous physical activity (MVPA) per week. The user was encouraged to set a goal corresponding to 30 min of daily MVPA, i.e., 210 min per week.

Users were asked to record all physical activities of at least moderate intensity every day. If the performed activity was perceived as vigorous, the user was instructed to double the number of minutes recorded. It was possible to record several bouts of activity during the same day and to add activity to previous days. A daily reminder to record activity was sent to everyone at 8 pm regardless if they already had recorded activity or not.

The individual weekly goal and the total minutes recorded each week of the intervention were illustrated by a graph in the smartphone application. On Sundays, users who reached their personal goal and/or had recorded at least 150 min of activity received an encouraging message telling them to keep up the good work during the upcoming week. Those who did not reach their goal received a message with encouragement to try again next week.

In addition to the physical activity component of the smartphone application, information regarding the health benefits of physical activity, medications, vitamin supplementation, and diet recommendations after surgery was also included. This information was based on the information given within standard care, but was here also made available within the app. Users received push messages with information and encouraging texts of different lengths connected to this information on a pre-determined schedule. The frequency of information messages was higher in the beginning of the intervention period with multiple messages per week and less frequent towards the end with a message every other week.

### Standard Care

Both groups received routine information, including general information on diet and post-operative physical activity, as a part of standard care. They all had pre-operative visits and post-operative visits at 6 weeks and 12 months after surgery. The control group did not receive any intervention or additional information on for example benefits of physical activity, medications, or vitamins, other than that routinely included in standard care. While weight is assessed at every post-operative visit, post-surgery physical activity is not evaluated in a standardized manner within standard care.

### Outcome Measures—Physical Activity

Physical activity was measured using the triaxial Actigraph wGT3x-BT accelerometer [23] pre-surgery (baseline) and at post-surgery follow-ups after 18 weeks and 6 and 12 months. Participants were asked to wear the accelerometer on their wrist during all hours of seven consecutive days. The accelerometer collected data at 80 Hz. Raw acceleration data were extracted through ActiLife version 6.13.3 and processed using open source R-package GGIR version 2.0-0 (https://cran.r-project.org/web/packages/GGIR/index.html). Data was aggregated through application of Euclidian norm minus one (ENMO), where negative values were rounded up to zero. Default settings were applied [24–26].

MVPA was calculated using the GGIR default cut point for the non-dominant wrist (100 m*g*) [27]. Similar to the Whitehall II study [28], MVPA was measured in bouts of at least 1 min with an 80% filter; i.e., 80% of the epochs had to be equal to or above the MVPA threshold. Default cut points were also used to define inactivity (< 40 m*g*) and LPA (40–100 mg). Inactivity was defined as bouts of at least 10 min with 90% filter, and LPA was defined as bouts of at least 1 min with 80% filter. All variables were weighted to consist of five parts of data collected during the weekdays and two parts during the weekend. A valid measurement was defined as at least 14 h wear time per day from at least 4 days, whereof at least 1 day during the weekend.

### Outcome Measures—Weight Loss

Weight was measured pre-surgery at baseline and at post-surgery follow-ups after 6 and 18 weeks and 6 and 12 months. Height was measured at baseline. BMI was calculated as weight in kilograms divided by squared length in meters (kg/m^2^). Percent total weight loss (%TWL) was calculated at follow-ups as lost weight at each time point divided by total body weight at baseline, multiplied by 100 to obtain percentage.

### Statistical Analysis

Characteristics are presented by study group as mean (SD) for continuous variables and *n* (%) for categorical variables. Differences between study groups were tested using independent *t*-tests or non-parametric Wilcoxon rank-sum test for continues variables and chi^2^ tests for categorical variables. We calculated Cohen’s *d* to estimate effect sizes for the difference between means between the control and intervention groups. We used linear mixed models with fixed and random intercept and slope for the time variables to assess if there were longitudinal differences in outcomes between the intervention and control groups. In addition to time and group terms, a group*time interaction term was included to assess if any differences in outcomes were constant at follow-ups. Analysis of intervention effect were made following the intention-to-treat approach [29] and missing data was assumed to be missing at random as drop-out rates were very low in both study groups. The degree of missing data differed between weight and physical activity outcomes, but was similar in both groups at each time point. A *p*-value < 0.05 was considered statistically significant. Statistical analysis was performed using STATA 14.2 (StataCorp LP).

## Results

Baseline characteristics of all participants randomized to intervention (*n* = 74) or control group (*n* = 72) are presented in Table 1. There were no statistically significant differences in characteristics between the groups. Most of the participants, 79.5% (116/146), were women. The mean age of all participants was 40.9 years, and the mean BMI was 40.5 kg/m^2^ and did not differ between women (40.4 kg/m^2^) and men (41.1 kg/m^2^) (*p* = 0.54). The majority of participants, 81.5% (119/146), had a gastric bypass procedure performed. There was no difference in the distribution of type of surgical procedure between the study groups (*p* = 0.47).

**Table 1:** Baseline characteristics by study group for all participants that were randomized (*n* = 146) in the PromMera study.

	Control group		Intervention group			
	(*n* = 72)		(*n* = 74)			
	Mean	(SD)	Mean	(SD)	*p*1	*d*^2^
Age, years	40.6	(9.5)	41.2	(10.1)	0.70	0.06
Body weight, kg	114.6	(17.2)	115.6	(20.3)	0.76	0.05
BMI, kg/m^2^	40.7	(5.7)	40.4	(5.6)	0.76	0.05
Inactivity, min/day	598	(174)	648	(157)	0.09	0.31
LPA, min/day	32.5	(18.1)	30.2	(21.8)	0.21	0.11
MVPA, min/day	43.2	(45.9)	30.2	(23.6)	0.10	0.36
	*n*	(%)	*n*	(%)		
Sex					0.75	
Female	58	(80.6)	58	(78.4)		
Male	14	(19.4)	16	(21.6)		
Type of surgery					0.47	
Gastric bypass	57	(79.2)	62	(83.8)		
Gastric sleeve	15	(20.8)	12	(16.2)		
Smoking^3^					0.59	
Yes	1	(1.4)	2	(2.7)		
No	66	(91.7)	69	(93.2)		
Occupation^4^					0.12	
Working	54	(75.0)	63	(85.1)		
Parental leave/studying/sick leave	17	(23.6)	10	(13.5)		
Level of education^5^					0.36	
≤ 9 years	5	(6.9)	7	(9.5)		
10–12 years	46	(63.9)	40	(54.1)		
> 12 years	19	(26.4)	27	(36.5)		
Medication/treatment for:						
Diabetes	6	(8.33)	7	(9.5)	0.81	
Hypertension	15	(20.8)	16	(21.6)	0.91	
Hyperlipidemia	3	(4.2)	3	(4.1)	0.86	
Sleep apnea (CPAP)	6	(8.3)	4	(5.4)	0.78	
Depression/anxiety	16	(22.2)	13	(17.6)	0.65	
Pain^6^	11	(15.3)	9	(12.2)	0.75	

^1^From *t*-test for continuous variables (age, body weight, BMI), Wilcoxon rank-sum test (inactivity, LPA, MVPA), and chi^2^ test for categorical variables; ^2^Cohen’s *d*; ^3^*n* = 8 missing; ^4^*n* = 2 missing; ^5^*n* = 2 missing ^6^Related to arthrosis or other musculoskeletal disorder

Abbreviations: *BMI* body mass index, *CPAP* continues positive airway pressure, *LPA* light physical activity, *MVPA* moderate-to-vigorous physical activity

All participants had data on weight at baseline and 78.1% (114/146) had accelerometer data. Participants lacking accelerometer data were statistically significantly younger than those that had data (mean age 36.1 vs 42.2 years, *p* = 0.002), but no other differences in baseline characteristics were seen. Median and mean values of inactivity, LPA, MVPA, BMI, and %TWL at baseline pre-surgery and follow-ups are shown in Table 2. At baseline, mean inactivity, LPA, and MVPA were 625, 31.3, and 36.2 min/day, respectively, among all participants. Six weeks after surgery, participants had lost on average 15.8% of their weight from baseline. There were no statistically significant differences in BMI or %TWL between the control and intervention groups at the 6-week follow-up.

**Table 2:** Summary of outcome variables at baseline pre-surgery and follow-ups after 6 weeks (weight outcomes only, start of intervention), 18 weeks, 6 months, and 12 months

	Control group (*n* = 72)^1^				Intervention group (*n* = 74)^1^					
*n*	Median	Mean	(SD)	*n*	Median	Mean	(SD)	*p*^*2*^	*d*^*3*^	
Inactivity, min/day										
Baseline	53	593	598	(174)	61	631	648	(157)	0.09	0.31
18 weeks	41	588	609	(149)	35	606	639	(168)		0.19
6 months	37	563	581	(157)	42	685	653	(192)		0.41
12 months	33	619	620	(174)	33	671	677	(152)		0.35
LPA^3^, min/day										
Baseline	53	34.5	32.5	(18.1)	61	24.8	30.2	(21.8)	0.21	0.11
18 weeks	41	31.1	35.6	(20.4)	35	26.1	34.0	(23.9)		0.07
6 months	37	37.8	42.3	(24.6)	42	29.3	37.2	(21.8)		0.22
12 months	33	33.2	42.1	(29.3)	33	35.0	42.9	(29.1)		0.03
MVPA^4^, min/day										
Baseline	53	31.5	43.2	(45.9)	61	25.8	30.2	(23.6)	0.10	0.36
18 weeks	41	25.1	36.5	(54.4)	35	33.3	40.9	(32.4)		0.10
6 months	37	32.9	45.3	(46.8)	42	35.2	40.6	(33.5)		0.12
12 months	33	30.1	38.9	(31.8)	33	33.9	38.9	(27.9)		0.002
BMI, kg/m^2^										
Baseline	72	39.3	40.7	(5.7)	74	39.3	40.4	(5.6)	0.76	0.05
6 weeks	70	33.3	34.1	(5.3)	73	33.5	34.2	(5.5)		0.03
18 weeks	51	28.8	30.3	(5.4)	57	30.6	31.3	(5.2)		0.19
6 months	57	28.5	28.7	(3.9)	59	27.9	29.5	(5.2)		0.16
12 months	68	26.6	27.0	(4.9)	72	26.7	27.6	(4.6)		0.13
%TWL^5^										
6 weeks	70	16.0	16.2	(3.5)	73	15.0	15.5	(3.4)	0.20	0.21
18 weeks	51	25.5	25.5	(5.1)	57	23.2	23.0	(5.2)		0.49
6 months	57	29.0	28.4	(5.0)	59	26.7	27.4	(4.6)		0.22
12 months	68	34.4	33.7	(6.4)	72	31.9	31.6	(6.2)		0.33

^1^At baseline; ^2^Student’s *t*-test comparing baseline levels of BMI and 6-week assessment of %TWL and Wilcoxon rank-sum test comparing baseline levels of inactivity, LPA, and MVPA between the study groups; ^3^Cohen’s *d*; ^4^accelerometer data available for *n* = 53 in the control group and *n* = 61 in the intervention group at baseline; ^5^weight data available for *n* = 70 in the control group and *n* = 73 in the intervention group at 6-week follow-up

Abbreviations: *BMI* body mass index, *LPA* light physical activity, *MVPA* moderate-to-vigorous physical activity, *TWL* total weight loss

**Fig. 2:**
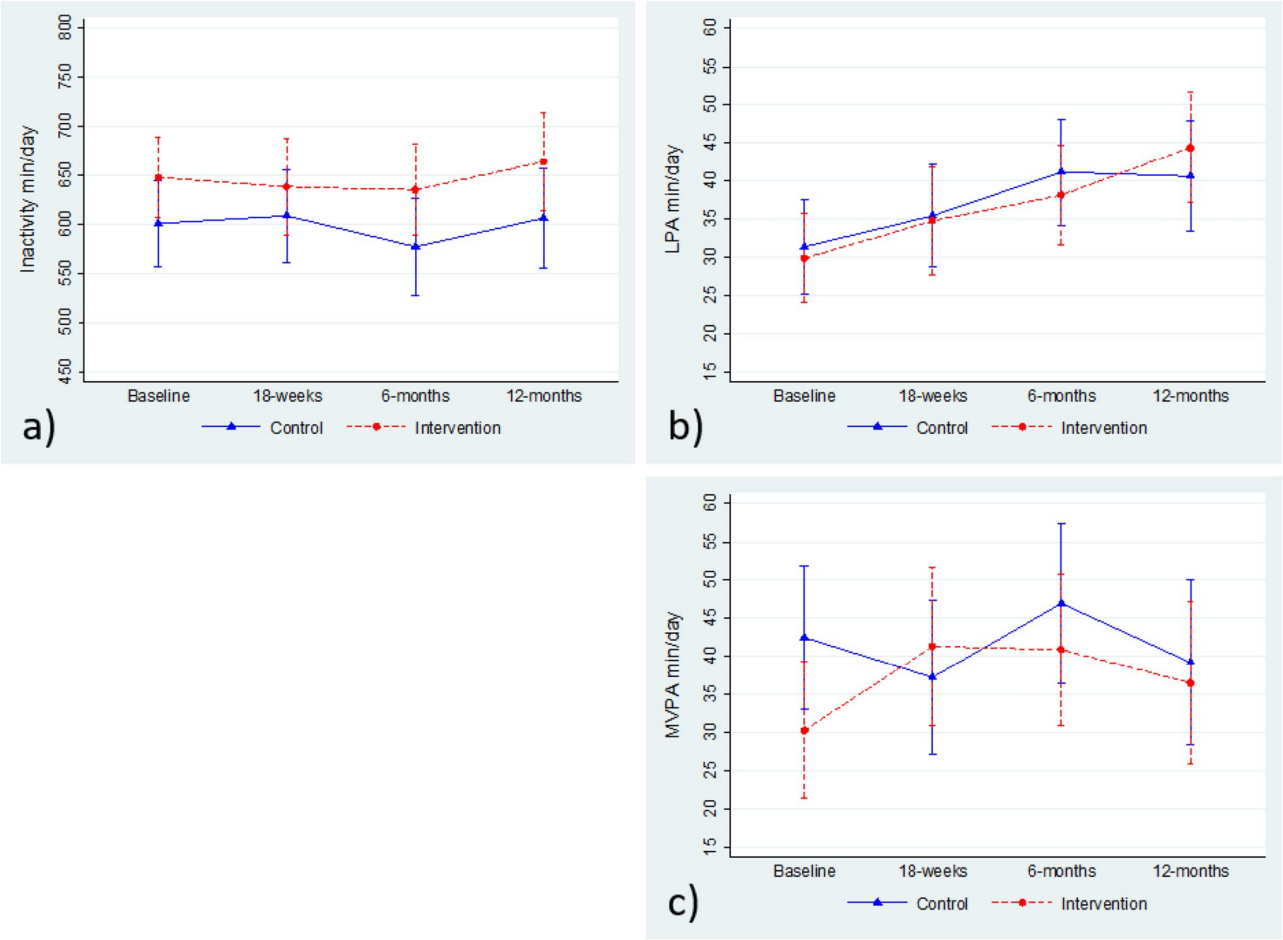
Changes over time in **a** inactivity (min/day), **b** light physical activity (LPA) (min/day), and **c** moderate-to-vigorous physical activity (MVPA) (min/day) in the control group and the intervention group. Group means with 95% CIs from linear mixed models are shown for each time point; results correspond to model estimates shown in Table 3

**Fig. 3:**
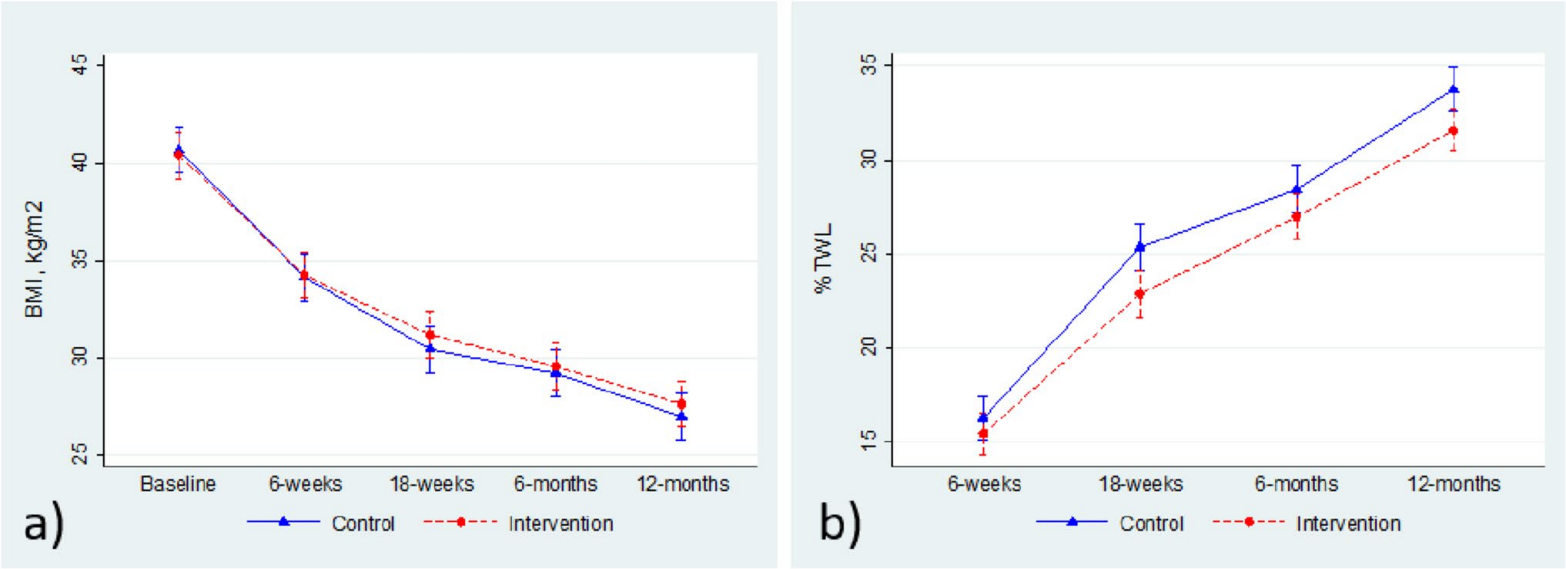
Changes over time in **a** body mass index (BMI) (kg/m^2^) and **b** percent total weight loss (%TWL), in the control group and the intervention group. Group means with 95% CIs from linear mixed models are shown for each time point; results correspond to model estimates shown in Table 3

Results of the intervention effect on inactivity, LPA, MVPA, BMI, and %TWL are shown in Table 3 and Figs. 2 and 3. We found a statistically significant effect of the intervention (group-by-time interaction 16.2, 95% CI 3.5 to 28.9) on MVPA at 18 weeks. This is resulting from the fact that the mean MVPA in the intervention group was lower at baseline than at 18-week follow-up (30.2 vs. 40.9 min/day), while the opposite was seen among controls that had higher MVPA at baseline (43.2 vs. 36.5 min/day). There was no statistically significant difference in mean MVPA min/day at 18 weeks between the groups and no intervention effect on MVPA at the 6- or 12-month follow-up. We found no effect of the intervention or differences in means at any follow-up for inactivity or LPA.

**Table 3:** The intervention effect on daily minutes of moderate-to-vigorous physical activity (MVPA, primary outcome) and inactivity, light physical activity (LPA), body mass index (BMI), and percent total weight loss (%TWL) in the PromMera study

	Model estimates1			
	Difference^2^		Group-by-time interaction	
	Mean	(95% CI)	*β*	(95% CI)
Inactivity, min/day				
18 weeks	29.8	(− 38.8 to 98.4)	− 17.4	(− 82.5 to 47.6)
6 months	58.0	(− 9.8 to 125.8)	10.8	(− 53.2 to 74.9)
12 months	57.6	(− 14.1 to 129.4)	10.4	(− 58.1 to 78.9)
LPA, min/day				
18 weeks	− 0.7	(− 10.4 to 9.1)	0.8	(− 9.0 to 10.6)
6 months	− 3.0	(− 12.6 to 6.6)	− 1.5	(− 11.2 to 8.1)
12 months	3.8	(− 6.4 to 14.0))	5.3	(− 5.1 to 15.6)
MVPA, min/day				
18 weeks	4.0	(− 10.5 to 18.6)	**16.2**	**(3.5 to 28.9)**
6 months	− 6.1	(− 20.4 to 8.3)	6.1	(− 6.4 to 18.6)
12 months	− 2.7	(− 17.8 to 12.4)	9.5	(− 4.0 to 22.9)
BMI, kg/m^2^				
6 weeks	0.1	(− 1.6 to 1.8)	0.4	(− 0.3 to 1.1)
18 weeks	0.8	(− 1.0 to 2.5)	**1.0**	**(0.3 to 1.8)**
6 months	0.3	(− 1.4 to 2.05)	0.6	(− 0.1 to 1.4)
12 months	0.7	(− 1.0 to 2.35)	**0.9**	**(0.3 to 1.6)**
%TWL				
18 weeks	− **2.5**	**(**− **4.3 to** − **0.7)**	− 1.7	(− 3.3 to 0.005)
6 months	− 1.5	(− 3.2 to 0.3)	− 0.6	(− 2.3 to 1.0)
12 months	− **2.2**	**(**− **3.8 to** − **0.5)**	− 1.4	(− 2.9 to 0.2)

^1^Results from linear mixed model (*n* = 121; inactivity, LPA, MVPA, and *n* = 146; BMI, %TWL); ^2^difference between groups at specified time point based on results from linear mixed models.

Values in bold indicate statistical significance

At follow-up after 18 weeks and 12 months, both groups had profound weight loss, but the control group had lowered their BMI significantly more than participants in the intervention group. However, there were no differences in the mean BMI between the groups at any follow-up. There were no statistically significant differences between the control group and the intervention group with regard to change in %TWL. There were also small, but statistically significant, differences in mean %TWL, favoring the controls, at follow-up after 18 weeks and 12 months.

## Discussion

In this randomized controlled trial targeting physical activity after bariatric surgery, we found no clear effect of using a smartphone application promoting increased activity. Although our results indicated an effect of the intervention on MVPA at follow-up after 18 weeks and on BMI at follow-up after 18 weeks and 12 months, the mean MVPA or BMI at the same time points did not differ between study groups. We found no evidence of an effect of the intervention on inactivity, LPA, or %TWL.

The statistically significant intervention effect seen for MVPA may be explained by a difference, although not statistically significant, in MVPA min/day at baseline between the groups, with higher MVPA in the control group. In part, that difference can be attributed to one participant in the control group having a very high level of MVPA (as indicated by the large SDs also), but even when excluding this participant, the significant effect of the intervention remains. An increase in MVPA was seen from baseline to the 18-week follow-up in the intervention group, while the control group decreased their MVPA during the same period, thereby resulting in a difference in change (i.e., effect), but no difference in mean MVPA at the follow-up. Levels of baseline MVPA in our study are similar to pre-surgery levels of MVPA seen in a previous study on the same patient group in Sweden [11].

It is possible that the period of active intervention was too short to create a lasting change. However, intervention periods of 8–12 weeks have previously shown positive effects [19]. A longer intervention period may lead to higher attrition or decreased compliance to the intervention over time. High attrition rates are a common problem in mHealth interventions and we have previously shown that approximately half of the participants stopped using the PromMera smartphone application during the active intervention [22]. This could partly explain why we did not see an effect in our study. The manual registration in the PromMera smartphone application may have impacted user-friendliness, and automatic registration of activities likely promotes continued usage and higher registration rates [30].

BMI at the 12-month follow-up in our study are very similar to the corresponding numbers reported for patients after bariatric surgery in Sweden [31]. Our results do not support additional effects on weight loss among patients receiving an intervention targeting physical activity. However, this may be explained by the lack of an effect on physical activity levels in the first year. Our study is limited by the fact that participants are only followed during the first year post surgery when the variability in %TWL is small. Studies with longer follow-up time are needed to investigate long-term effects. Post-operative exercise has previously been associated with increased weight loss in systematic reviews of observational studies [1–3], and one systematic review and meta-analysis of randomized controlled trials targeting post-bariatric surgery exercise showed positive results [4]; another did not [5].

Strengths of our study are the randomized controlled study design and the large sample size. We recruited participants from one hospital operating patients from an entire health care region, including both rural and urban areas. Our study population was comparable to Swedish bariatric surgery patients in 2018 with regard to age, BMI, sex, and educational level [32]. The inclusion criteria of having access to and ability to use a smartphone could potentially introduce selection of study participants. However, given that more than 90% of all adults in Sweden were smartphone users, independent of socio-economic status, in 2018 [33], we believe that the risk of selection bias due to this is very low.

The objective assessment of outcome variables is also a strength. Physical activity was objectively measured using accelerometers which are likely to provide a more valid estimate of physical activity compared to self-reported data. However, to increase compliance to accelerometer measurements, our participants were instructed to wear the accelerometer on the wrist. This is not as commonly used as on the hip, and therefore, the cut points for MVPA are less established. A limitation is also that we did not instruct participants to wear the accelerometer on their non-dominant wrist, which potentially could have led to slightly different assessments of physical activity, since wearing on the dominant wrist may overestimate MVPA. Some argue that there is high agreement between dominant- and non-dominant wrist variable outputs when using GGIR [34], while others disagree [35].

In conclusion, our results indicate that a smartphone application targeting primarily physical activity may have the potential to promote short-term MVPA post bariatric surgery. The use of new digital solutions, including smartphone applications to promote physical activity, that allow for easily accessible, scalable, and individually tailored interventions should be further evaluated within this patient group.
